# Design and implementation of questionnaires to assess chronic exposure to secondhand smoke in children and adults

**DOI:** 10.1186/1617-9625-12-S1-A28

**Published:** 2014-06-06

**Authors:** Mary Misailidi, Christos I Papakonstantinou, Manolis N Tzatzarakis, Mathaios P Kavvalakis, Yiannis Koutedakis, Aristidis M Tsatsakis, Andreas D Flouris

**Affiliations:** 1 FAME Laboratory, Institute of Research and Technology Thessaly, Centre for Research and Technology Hellas, Trikala, 42100, Greece; 2 Centre of Toxicology Science and Research, School of Medicine, University of Crete, Heraklion, 71003, Greece; 3 Department of Exercise Sciences, University of Thessaly, Trikala, 42100, Greece

## Background

Despite a multitude of anti-smoking campaigns being active worldwide, the number of smokers is currently larger than at any other time in human history. An essential first step towards minimizing the health effects of secondhand smoke (SHS) is to accurately assess the exposure level of individuals. The objectives of this study were to: (i) to develop questionnaires that can identify never-smoking children and adults experiencing increased exposure to secondhand smoke (SHS+), (ii) to determine their validity against hair nicotine, and (iii) assess their reliability.

## Materials and methods

A sample of 191 children (85 males; 106 females; 7-18 years) and 95 adult (23 males; 72 females; 18-62 years) never-smokers consented to hair nicotine analysis and answered a large number of questions pertaining to all relevant sources of SHS. A randomly-selected 30% answered the questions again after 20-30 days.

## Results

Prevalence of SHS+ in children and adults was 0.52±0.07 and 0.67±0.10, respectively (p<0.05). The Smoke Scale for Children (SS-C) and the Smoke Scale for Adults (SS-A) were developed via factor analysis and included nine questions each. Positivity criteria for SS-C and SS-A via receiver operating characteristics (ROC) curve analysis were identified at >16.5 and >16, respectively. Significant Kappa agreement (p<0.05) was confirmed when comparing the SS-C and SS-A to hair nicotine concentration. Reliability analyses demonstrated that the SS-C and SS-A scores obtained on two different days are highly correlated (p<0.001) and not significantly different (p>0.05). Area under the curve and McNemar's Chi-square showed no pair-wise differences in sensitivity and specificity at the cutoff point between the two different days for SS-C and SS-A (p>0.05).

## Conclusions

We conclude that the SS-C and the SS-A represent valid, reliable, practical, and inexpensive instruments to identify children and adult never-smokers exposed to increased SHS.

